# A Meta-Analysis of the Association between Gender and Protective Behaviors in Response to Respiratory Epidemics and Pandemics

**DOI:** 10.1371/journal.pone.0164541

**Published:** 2016-10-21

**Authors:** Kelly R. Moran, Sara Y. Del Valle

**Affiliations:** Analytics, Intelligence and Technology Division, Los Alamos National Laboratory, Los Alamos, New Mexico, United States of America; Hokkaido University Graduate School of Medicine, JAPAN

## Abstract

Respiratory infectious disease epidemics and pandemics are recurring events that levy a high cost on individuals and society. The health-protective behavioral response of the public plays an important role in limiting respiratory infectious disease spread. Health-protective behaviors take several forms. Behaviors can be categorized as pharmaceutical (e.g., vaccination uptake, antiviral use) or non-pharmaceutical (e.g., hand washing, face mask use, avoidance of public transport). Due to the limitations of pharmaceutical interventions during respiratory epidemics and pandemics, public health campaigns aimed at limiting disease spread often emphasize both non-pharmaceutical and pharmaceutical behavioral interventions. Understanding the determinants of the public’s behavioral response is crucial for devising public health campaigns, providing information to parametrize mathematical models, and ultimately limiting disease spread. While other reviews have qualitatively analyzed the body of work on demographic determinants of health-protective behavior, this meta-analysis quantitatively combines the results from 85 publications to determine the global relationship between gender and health-protective behavioral response. The results show that women in the general population are about 50% more likely than men to adopt/practice non-pharmaceutical behaviors. Conversely, men in the general population are marginally (about 12%) more likely than women to adopt/practice pharmaceutical behaviors. It is possible that factors other than pharmaceutical/non-pharmaceutical status not included in this analysis act as moderators of this relationship. These results suggest an inherent difference in how men and women respond to epidemic and pandemic respiratory infectious diseases. This information can be used to target specific groups when developing non-pharmaceutical public health campaigns and to parameterize epidemic models incorporating demographic information.

## Introduction

### Motivation and overview

Respiratory infectious disease pandemics are unpredictable yet recurring events that levy a high cost on individuals and society. Throughout history, respiratory disease epidemics and pandemics have imposed a severe worldwide cost. Of the 8,098 people worldwide who became sick with SARS, 774 died during the 2003 outbreak [1], and since 2003 about 60% of the 650 people that have been infected with highly pathogenic H5N1 avian influenza have died from their illness [2]. During the 2009 influenza A (H1N1) pandemic, there were over 60 million cases and 12,000 deaths in the United States alone [3], and an estimated 201,200 respiratory deaths globally [4].

Pharmaceutical interventions alone cannot be relied upon to stem the tide of pandemic outbreaks. While influenza transmission can be halted with the use of antiviral medications, mutations in the virus necessitate that a new vaccine be produced for each new flu strain. Vaccination production can take up to six months to complete, with the burdens of delays, likely shortages, and virus mismatch reducing the potential impact of the vaccine. Furthermore, pharmaceutical interventions often require consultation with a physician or, in more severe cases, hospitalization. These requirements reduce the potential impact of pharmaceutical interventions due to the fact that many people do not have access to health care or refuse to be seen by a health care provider. Additionally, it is often impossible to satisfy this requirement during a pandemic influenza outbreak because the demand for staff, facilities, and equipment often exceeds the supply [5]. The limitations of pharmaceutical interventions during pandemic influenza outbreaks highlight the importance of also incorporating non-pharmaceutical interventions in public health campaigns aimed at limiting respiratory infectious disease spread.

The success of campaigns designed to limit disease transmission relies on the public’s protective behavioral responses to an epidemic/pandemic. Some of these responses are individual responsibilities, while others deal with compliance to governmental mandates or laws. Protective behaviors can be broadly grouped into three categories: preventive, avoidant, and management [6]. Preventive behaviors may be non-pharmaceutical measures (e.g., hand washing, sanitation, and mask wearing) or pharmaceutical measures (e.g., vaccination uptake). Avoidant behaviors include staying home from work or school, avoiding public or crowded settings, and complying with quarantine constraints, all examples of non-pharmaceutical measures. Management behaviors include taking pharmaceutical antiviral medications and seeking medical help. More examples of health-protective behaviors are shown in Table 1. An understanding of the demographic determinants of protective human behavior can inform the communication strategies of both pharmaceutical and non-pharmaceutical interventions during an epidemic/pandemic disease outbreak.

**Table 1:** Examples of non-pharmaceutical and pharmaceutical health-protective behaviors. Note that this table represents examples of each type of behavior rather than a comprehensive list of all behaviors included in this analysis.

Behavior type	Non-pharmaceutical behaviors	Pharmaceutical behaviors
**Preventive**	Hand washing Using tissues when coughing or sneezing Face mask wearing Surface cleaning	Vaccination
**Avoidant**	Avoiding crowds Avoiding public transit Quarantine compliance Staying home from school/work	
**Management**	Seeking professional medical advice Using Internet or phone help resources	Tamiflu use

In addition, determining the drivers that are responsible for these protective behaviors can inform mathematical models that are intended to provide decision support. Mathematical modeling is typically used to understand disease dynamics and assess the impact of different interventions [7, 8]. Recently, several models [9–12] have attempted to include human behavior but their impact has been limited by the lack of quantitative consensus regarding how behavior is influenced by different demographic characteristics. As such, understanding these behavioral factors is crucial for devising public health campaigns, providing information to parametrize mathematical models, and ultimately limiting disease spread.

### Related work

A 2010 review paper by Bish and Michie [6] identified several demographic determinants associated with a higher probability of adopting protective behaviors during a pandemic, including being older, female, more educated, and non-White. However, this review offers a qualitative rather than quantitative analysis, providing a discussion of the different factors rather than a comprehensive investigation of the data presented in each of the studies. This qualitative assessment lacks a definitive conclusion due to the equivocal conclusions reached by the individual articles reviewed. While the authors find that “when there is a significant difference women are consistently more likely than men to carry out the behaviors,” they note that some studies find no gender differences.

With regard to the association between gender and pharmaceutical interventions, a qualitative conclusion was reached in a 2011 systematic review paper by Bish, et. al [13]. The authors found that in the general population men were more likely to intend to be vaccinated and to be vaccinated than women. However, the authors note two studies for which this relationship is not present.

A 2011 Ph.D. thesis by Liao [14] included a section focused on the demographic determinants of individuals adopting protective behaviors in the context of a pandemic outbreak. Liao’s review finds that women consistently report more adoption of hygiene practices, government-recommended behaviors, and avoidance behaviors, and men regularly have higher vaccination intention. However, several studies find no association between gender and protective behavior. As in [6] and [13], the conclusion reached in Liao’s review is qualitative rather than quantitative and a deeper statistical understanding of determinants of human behavior in the context of pandemic outbreaks is not reached. Furthermore, in each review discussed thus far the sample size of studies considered numbered less than twenty for gender analysis.

### Current analysis

In this paper, a meta-analysis is performed in order to quantitatively analyze the body of scholarly work relating to demographic determinants of human behavior in the context of epidemic and pandemic respiratory diseases, specifically avian influenza, swine influenza, Middle East respiratory syndrome (MERS), and severe acute respiratory syndrome (SARS). To our knowledge, no previous study has quantified the direction and magnitude of the relationship between demographic characteristics and protective behavior in the general population in response to respiratory epidemics/pandemics. Potential moderating influences of study characteristics on this relationship are also tested. The results of this analysis will inform decision makers, public health officials, and modelers as to the differing behavioral response by men and women during epidemic and pandemic respiratory disease outbreaks.

Meta analyses are ideal for research areas in which the body of literature addressing a shared research hypothesis is saturated but the conclusion is still unclear [15]. Compared to traditional or systematic reviews, such as that of Bish and Michie [6], meta-analyses have the advantage of being reproducible, able to include studies for which the results lack statistical significance, and able to quantify both the magnitude and the significance of the relationship in question. The disadvantage of meta-analyses compared to narrative reviews is that they cannot cover the same breadth of topics. While Bish and Michie [6] addressed age, gender, ethnicity, educational level, working status, marital status, and psychological factors associated with protective behaviors, this study covers only the association between gender and protective behaviors.

## Methods

### Literature search strategy

**Fig 1:**
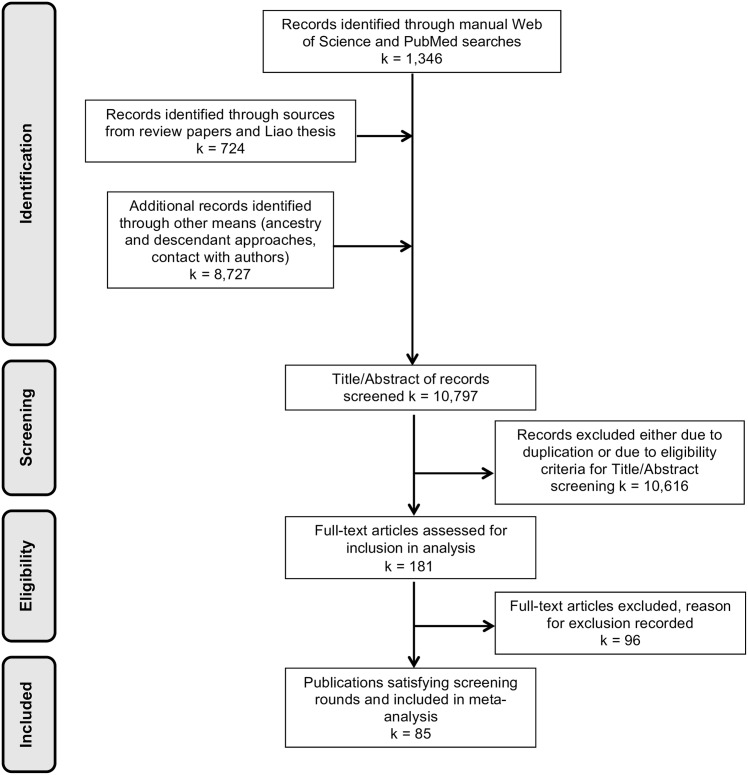
Preferred Reporting Items for Systematic Reviews and Meta-Analyses (PRISMA) flow diagram of search strategy. A flow diagram providing the organization of the article search and selection process along with values for article retention numbers at each state.

The recommendations outlined in [16] were followed when carrying out the article search and selection process. A flow diagram of the article search and screening process is shown in Fig 1. Web of Science and PubMed databases were searched from 25 to 31 August of 2015 for relevant articles using the search queries identified in S1 Table. Records were also identified through sources from relevant review papers and Liao’s 2011 thesis: see Refs. [6, 13, 17–23] for all review articles used. After these initial records were screened and relevant articles were chosen to be considered in full text screening, further records were identified through ancestry and descendant approaches. That is, searches for papers either citing or cited by these sources, using all of these initially identified articles.

A challenge to performing a successful meta-analysis is publication bias and associated dissemination biases [24]. In an attempt to identify any unpublished works on the topic, records were also sought out through contact with the authors of the review papers and theses used in the article search process, Refs. [6, 13, 17–23], requesting leads on any unpublished or in-progress studies. These authors were chosen as contacts because they are assumed to be well acquainted with the current state of topic literature. Although contact with researchers yielded no additional records, publication bias is of minimal concern. Studies on behavioral response to epidemics/pandemics usually address multiple possible factors. It is a plausible assumption that the lack of significance of one particular factor, in this case gender, would not keep a study from being published.

### Eligibility criteria

The inclusion and exclusion criteria for studies in this analysis were:

Inclusion (all criteria required)

1. Population: general population. Studies could focus on age- or location-specific subsets of the general population, such as college students, the elderly, or dwellers of a particular city. However, neither patient groups (e.g., HIV-positive or diabetic individuals), nor population groups based on special qualities (e.g., parents or pregnant women), nor travelers (e.g., groups sampled at airports or Hajj pilgrims), nor specific professional groups (e.g., healthcare workers or teachers) could be the focus of the study.
2. Diseases: epidemic and pandemic respiratory infectious diseases. Namely, avian influenza, swine influenza, Middle East respiratory syndrome (MERS), and severe acute respiratory syndrome (SARS).
3. Behaviors: preventive, avoidant, and management health-related behaviors (reported, intended, or actual).
4. Demographic characteristics: gender included. The association between gender and the addressed behavior had to be reported in the context of the aforementioned epidemic/pandemic respiratory diseases.
5. Date: published from 2002 to present.
6. Language: published in the English language.

Exclusion (each criterion can exclude)

1. Type of study: qualitative or focus group studies, mathematical modeling studies, studies about efficacy of behavioral interventions, studies about health policy.
2. Data: data describing the association between demographic traits and behavior were unavailable or unable to be converted into a log odds ratio.
3. Behavior: reported behavior involves animal-related behavior (e.g., chicken purchasing or cleaning behavior, bird or camel avoidance, etc.) or purchasing behavior (e.g., purchasing hand sanitizer or cleaning supplies).

### Study selection

Articles were screened against the above eligibility criteria at two stages: titles/abstract and full text. At the first stage, one reviewer (KM) screened the titles and abstracts of all identified texts for relevance. At the second stage, two reviewers (KM and SD) independently examined the full-text articles of all remaining records for eligibility based on the above inclusion and exclusion criteria. The second screening was duplicated in order to minimize selection bias. Discrepancies in exclusion choices were discussed and a final decision was made based on the criteria. The rationale for exclusion was recorded when a full-text article was reviewed and deemed unsuitable.

### Data extraction and coding

The aim of the data extraction process was to capture the association between gender and health-protective behavior in each study and to record any potential moderators (i.e., study-level variables that may influence the outcome) of this relationship. The term study is used as in [15] to describe a set of data collected under a single research plan from a designated sample of respondents. Under this definition, it is possible for one publication to present the results from several studies or for one study to be described in multiple publications. When two or more publications assessed the same study population, a joint study moniker was created and the relevant data from each publication were recorded under the moniker.

Both qualitative and quantitative data were extracted from all included studies. One reviewer (KM) recorded all relevant qualitative and quantitative data from each publication. The second reviewer (SD) cross-referenced the data with the text of each publication to check for accuracy and completeness. Reviewers resolved discrepancies between entries by discussion and consultation of the publication’s full text.

Publications commonly stated the association between gender and health-protective behavior as a count, percentage, or odds ratio for males and females adopting or increasing a given behavior. Less commonly, publications reported mean behavior scores and their associated standard deviations by gender. Counts, percentages, odds ratios, and mean behavior scores were all converted to the same effect size, namely log odds ratios and their associated log standard errors [25]. For cases in which relevant findings were reported but an effect size could not be calculated (e.g., publications that included gender in multiple regression equations or hierarchical linear models for behavior), the direction of the relationship was noted for comparison to analysis results. While new techniques have been developed for synthesizing variables from complex models, such techniques are still not widely used or agreed upon [26]. Although the results from complex models are not explicitly included in our meta-analysis, the trends captured by publications using these models are noted in the results section. These trends were recorded in order to compare the distribution of the direction of effects for these complex models to the distribution of the direction of effect sizes used in analysis.

For studies that did not include results on the basis of nonsignificance, the primary authors of these publications were contacted requesting results and associated error terms. In the case of nonresponse, missing nonsignificant results were excluded from the analysis. Although missing nonsignificant results may be treated as a perfect null value, meaning an effect size of zero, this approach is adequate only for rejecting the null hypothesis that gender plays no role in behavioral response and not for answering questions about size of effect and effect moderators [15]. Sensitivity analysis was performed to assess whether study results differed if missing nonsignificant results were treated as zeros rather than excluded.

Potential moderators recorded during data extraction were study design, country, mean age of sample population, behavior, behavior type (intended, reported, actual), behavior category (preventive, avoidant, management), pharmaceutical or non-pharmaceutical status, and overall percentage of respondents adopting/increasing the assessed protective behavior.

Both reviewers kept a log of the article search, identification, selection, data extraction, and coding process in order to maintain transparency and consistency. All review procedures followed Preferred Reporting Items for Systematic Reviews and Meta-Analyses (PRISMA) guidelines [27], and a complete PRISMA checklist is provided in S1 Fig.

### Statistical techniques

Since many of the included studies generated more than one relevant effect size (i.e., they addressed more than one behavior), an overall mean effect size for each study was constructed to ameliorate the issue of statistical dependence of the data points. If both pharmaceutical and non-pharmaceutical measures were addressed in a single study, mean effect sizes were also calculated for each of these types of behavior to use in separate analyses. For example, if a single study addressed hand washing, crowd avoidance, and vaccination, then the weighted average of the log odds ratios for hand washing and crowd avoidance would provide the non-pharmaceutical study metric, the log odds ratio for vaccination would provide the pharmaceutical study metric, and the weighted average of all three log odds ratios would provide the overall study metric. The weighted average of the effect sizes for *n* behaviors, ${\bar{y}}_{i}$, is given by

$${\bar{y}}_{i}=\frac{\sum w_{i}b_{i}}{\sum w_{i}},$$ (1)

where *b*_*i*_ and *w*_*i*_ represent the log odds ratio and weight, respectively, corresponding to behavior *i*. Inverse variance weights were used, i.e., $w_{i}=\frac{1}{v_{i}}$. Calculations are performed according to [28], and the relevant code used for these calculations can be found in S2 Code. Upon generating these weighted averages for all studies addressing multiple behaviors, three datasets were formed:

1. The set of studies addressing non-pharmaceutical behaviors, each providing a log odds ratio that is, for studies including multiple non-pharmaceutical behaviors, an average of the log odds ratios corresponding to each of those behaviors.
2. The set of studies addressing pharmaceutical behaviors, each providing a log odds ratio that is, for studies including multiple pharmaceutical behaviors, an average of the log odds ratios corresponding to each of those behaviors.
3. The full set of all studies, each providing a log odds ratio that is, for studies including multiple health-protective behaviors, an average of the log odds ratios corresponding to each of those behaviors.

It was assumed that pharmaceutical behaviors and non-pharmaceutical behaviors have their own distinct underlying distribution of effect sizes. Therefore, the analyses were performed independently for the set of effect sizes for pharmaceutical behaviors and the set of effect sizes for non-pharmaceutical behaviors. The analyses were also performed on the full set of effect sizes for all behaviors.

The order of procedures performed in the analysis followed recommendations by [15]. Prior to performing any calculations, outliers amongst independent study-level effect sizes were identified by examining the distribution of effect sizes and removing those with an effect size greater than three standard deviations from the mean. Further analysis proceeded using both the trimmed and untrimmed distributions, and the results were compared in a sensitivity analysis. All analyses were conducted using the metafor package [29] in R [30].

A fixed effects model was constructed following guidelines in [29], given by

$$y_{i}=\theta_{i}+e_{i}.$$ (2)

Let *k* equal the number of studies considered in the analysis. Each of the *i* = 1, …, *k* independent effect size observations are denoted by *y*_*i*_, with associated sampling variance *v*_*i*_. Each effect size is assumed to differ from its corresponding true effect size, denoted *θ*_*i*_, by a sampling error *e*_*i*_ ∼ *N*(0, *v*_*i*_). The model was fit using weighted least squares in order to provide an inference about the magnitude of the average true effect, ${\bar{\theta }}_{w}$, of the set of studies included in the analysis. The value of ${\bar{\theta }}_{w}$ is given by

$${\bar{\theta }}_{w}=\frac{\sum w_{i}\theta_{i}}{\sum w_{i}}.$$ (3)

Inverse variance weights were used, i.e., $w_{i}=\frac{1}{v_{i}}$. A confidence interval on ${\bar{\theta }}_{w}$ is given by

$$\left({\bar{\theta }}_{lower},{\bar{\theta }}_{upper}\right)=\left({\bar{\theta }}_{w}-z_{\left(1-\alpha \right)}\sqrt{\frac{1}{\sum w_{i}}},{\bar{\theta }}_{w}+z_{\left(1-\alpha \right)}\sqrt{\frac{1}{\sum w_{i}}}\right),$$ (4)

where *z*_1−*α*_ is the critical *z*-value representing the desired confidence level.

Homogeneity analysis was performed on the study effect sizes. In a homogeneous distribution, the various effect sizes that are averaged into a mean value all estimate the same population effect size and individual study effect sizes differ from the population effect size only through random sampling error [15]. The test for homogeneity relies on the *Q*-statistic,

$$Q=\sum w_{i}{\left(\theta_{i}-{\bar{\theta }}_{w}\right)}^{2},$$ (5)

which is distributed as a chi-square with *k-1* degrees of freedom. In a heterogeneous distribution, the value for Q will exceed the critical value for a chi-square with *k-1* degrees of freedom and the null hypothesis of homogeneity is rejected.

A random effects model was built to account for additional between-study variability beyond sampling error [29]. The true effects of sample studies are assumed to be composed of some unknown average true population effect size *μ* along with normally distributed deviation *u*_*i*_ ∼ *N*(0, *τ*^2^), i.e., *θ*_*i*_ = *μ* + *u*_*i*_. Eq (2) then becomes

$$y_{i}=\mu +u_{i}+e_{i}.$$ (6)

The average true effect *μ* was estimated and restricted maximum-likelihood estimation (REML) was used to estimate the total amount of heterogeneity among the true effects *τ*^2^. The *I*^2^ statistic, which estimates what percent of the total variability in effect size estimates is due to heterogeneity among the true effects, is reported in the results.

A mixed effects model was built to determine the amount of heterogeneity among the true effects accounted for by systematic between-study differences rather than immeasurable study differences or random variance [29]. In a mixed effects model the true effect size is given by

$$\theta_{i}=\beta_{0}+\beta_{1}x_{i1}+...+\beta_{n}x_{in}+u_{i},$$ (7)

where *x*_*ij*_ denotes the value of the *j*th moderator variable for the *i*th study. Eq (6) then becomes

$$y_{i}=\beta_{0}+\beta_{1}x_{i1}+...+\beta_{n}x_{in}+u_{i}+e_{i}.$$ (8)

Moderators were systematically tested to assess their responsibility for between-study variability. Moderators were first tested individually for significance. Individually significant moderators were combined in one model that was then tested for significance. Total heterogeneity was assessed using the REML approach. The following moderators were explored: study design, continent, culture, country development, mean age of sample population, behavior, behavior type (intended, reported, actual), behavior category (preventive, avoidant, management), and overall percentage of respondents adopting/increasing the assessed protective behavior.

Sensitivity analyses were performed on each set of studies in three ways: by removing effect size outliers, by using the trim-and-fill method, and by including studies whose results weren’t reported on the basis of non-significance. The first sensitivity analysis was performed by removing studies whose effect size was over three standard deviations from the mean effect size for that dataset. The second sensitivity analysis was performed using the trim and fill method in order to assess the potential effect that missing studies may have had on the observed result [24]. The trim-and-fill method augments each set of studies under the assumption that values possibly missing due to publication bias can be imputed based on the distribution of standard errors associated with the given effect sizes. This method relies on scrutiny of a funnel plot for assumed bias-induced asymmetry, which, if present, is corrected through the addition of results that lead to a visually symmetric funnel plot. The final sensitivity analysis included results that were mentioned in a study but for which an effect size value was unreported because of non-significance. Effect sizes were assigned a log odds ratio of 0 and log standard errors were imputed from similar studies following suggestions included in [31].

## Results

### Study characteristics

As shown in Fig 1, the literature search identified 10,797 records. A total of 10,616 records, including duplicates, were excluded during the initial screening and 96 publications were excluded during the full text screening. The most common reason for exclusion during full text screening was that the association between demographic traits and behavior was not reported. See Table 2 for the full distribution of reasons for exclusion. The level of agreement between the two reviewers (KM and SD) following the second round of screening was 88%, with 100% agreement reached following discussion of inconsistencies.

**Table 2:** Reasons for exclusion and their associated frequency in full text screening.

Reason for exclusion	Frequency
Demographic association not reported	32
Study addresses profession- or risk- specific subset of population	18
Results could not be converted into an effect size	16
Behavior studied in the context of seasonal rather than pandemic influenza	7
No behavioral response provided that is suitable for inclusion	7
Sample populations and measured behavioral response are same as another study	6
Uninterpretable or inconsistent results	6
Interview/focus group study	2
All results unreported due to nonsignificance	1
Duplicate record	1

There were 16 studies with results that could not be converted into a log odds ratio but for which the direction of the association between gender and behavior was available: see Refs. [32–47]. All 10 of the studies addressing non-pharmaceutical behaviors showed that females were more likely to increase or adopt the given behavior. Of the 5 studies addressing pharmaceutical behaviors, 3 showed that males were more likely to increase or adopt the given behavior. Only one study addressed both pharmaceutical and non-pharmaceutical behaviors, and it showed a positive relationship for the female gender and adoption of the given behaviors.

**Fig 2:**
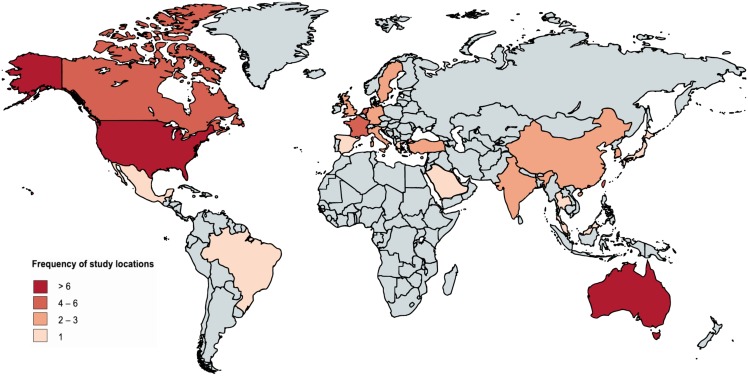
Map of global study distribution. A map visualizing the number of studies addressing populations from each country.

In total 85 publications satisfied all of the eligibility criteria and were included in the analysis (see Refs. [48–132]), with 88 independent study populations identified across these 85 publications. Of the included publications, 19 sampled populations in North America, 37 in Asia, 24 in Europe, 1 in Africa, 1 in South America, and 7 in Australia (note that some publications assessed multiple populations). The most commonly sampled countries were Hong Kong (18 studies), the United States (14 studies), and Australia (7 studies). See Fig 2 for a map showing the distribution of publication locations. The most common behaviors addressed in these studies included vaccination, avoidance behaviors, hand washing, and face mask use. Of the publications selected, 42.4% addressed pharmaceutical interventions and 37.6% addressed non-pharmaceutical interventions, with 20% addressing both. The full list of included publications and their relevant qualitative properties can be found in S2 Table.

**Fig 3:**
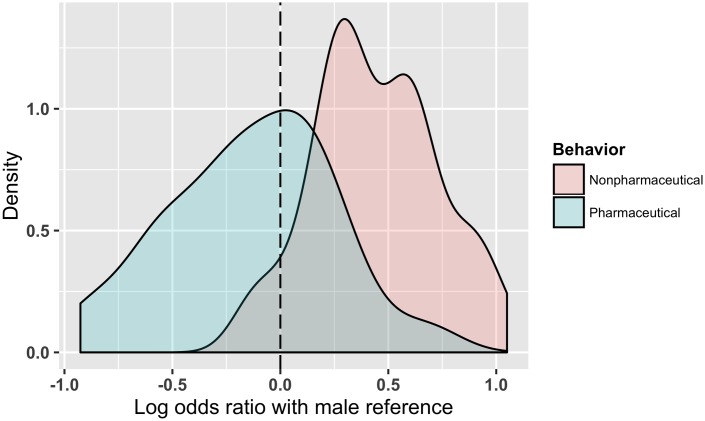
Density graph showing the sets of log odds ratios for pharmaceutical and non-pharmaceutical behaviors addressed for the 88 included study populations. Males are used as the reference; positive log odds ratios correspond to females being more likely to adopt/practice a given behavior, and negative log odds ratios correspond to males being more likely to adopt/practice a given behavior. The set of non-pharmaceutical behaviors shown is trimmed such that the log odds ratio falling outside of three standard deviations from the mean is excluded.

A density graph is shown in Fig 3 illustrating the effect sizes for the sets of pharmaceutical and non-pharmaceutical behaviors addressed for the 88 study populations in the analysis. In the following sections, the effect sizes of both types of behaviors are analyzed independently, and results for combined behaviors are available in S1 Results. The quantitative study-level data used throughout these sections can be seen in S1 Data.

### Fixed-effects models

A fixed-effects model was fitted to each of the sets of log odds ratios in order to determine the average true effect of the k studies included in each set. The set of 50 studies addressing non-pharmaceutical behaviors had an average log odds ratio of ${\bar{\theta }}_{w}=0.340$ (95% CI 0.318 to 0.363) and the set of 47 studies addressing pharmaceutical behaviors had an average log odds ratio of ${\bar{\theta }}_{w}=0.104$ (95% CI 0.103 to 0.105), implying that women were more likely than men to adopt/practice both pharmaceutical and non-pharmaceutical behaviors across the studies included in the analysis. Fixed-effects model results for the non-pharmaceutical and pharmaceutical sets are shown in Table 3. Results for the full study set are available in S1 Results.

**Table 3:** Fixed- and random-effects model results. Includes the non-pharmaceutical and pharmaceutical study sets and the three corresponding sensitivity analysis sets for each.

	**Non-pharmaceutical study set**			
	Original (k = 50)	Outlier removal (k = 49)	Trim-and-fill (k = 59)	With unreported (k = 51)
**Fixed-effects model**				
${\bar{\theta }}_{w}$ *(95% CI)*	0.340 (0.318 to 0.363)	0.351 (0.329 to 0.374)	*	0.331 (0.308 to 0.353)
*Q (p-val)*	314.930 (<0.0001)	192.145 (<0.0001)	*	314.620 (<0.0001)
**Random-effects model**				
*μ (95% CI)*	0.402 (0.307 to 0.496)	0.423 (0.356 to 0.490)	0.320 (0.223 to 0.418)	0.381 (0.289 to 0.473)
*τ*^2^ *(95% CI)*	0.093 (0.060 to 0.166)	0.038 (0.023 to 0.085)	0.119 (0.081 to 0.204)	0.090 (0.059 to 0.162)
*I*^2^ *(95% CI)*	92.74% (89.15 to 95.80%)	84.03% (76.47 to 92.21%)	93.49% (90.70 to 96.11%)	92.63% (89.11 to 95.76%)
	**Pharmaceutical study set**			
	Original (k = 47)	Outlier removal (k = 47)	Trim-and-fill (k = 51)	With unreported (k = 51)
**Fixed-effects model**				
${\bar{\theta }}_{w}$ *(95% CI)*	0.104 (0.103 to 0.105)	**	*	0.104 (0.103 to 0.105)
*Q (p-val)*	1835.407 (<0.0001)	**	*	1840.086 (<0.0001)
**Random-effects model**				
*μ (95% CI)*	-0.114 (-0.212 to -0.016)	**	-0.071 (-0.175 to 0.034)	-0.102 (-0.191 to -0.013)
*τ*^2^ *(95% CI)*	0.090 (0.058 to 0.177)	**	0.113 (0.076 to 0.223)	0.080 (0.053 to 0.157)
*I*^2^ *(95% CI)*	99.78% (99.66 to 99.89%)	**	99.81% (99.72 to 99.90%)	99.73% (99.59 to 99.86%)

${\bar{\theta }}_{w}$: Average true effect of the set of studies included in the analysis.

*Q*-statistic: Measure of heterogeneity. Calculated as the weighted sum of squared differences between individual study effects and the pooled effect across studies, with the weights being those used in the pooling method chi-square statistic with k-1 degrees of freedom *μ*: Average true population effect size.

*τ*^2^: Total amount of heterogeneity among the true effects.

*I*^2^: Percent of the total variability in effect size estimates due to heterogeneity among the true effects.

* Trim-and fill analysis results for fixed-effects model not relevant due to non-homogeneous effect size distribution.

** Results not shown because study set unchanged from original.

### Homogeneity analysis and random effects models

For homogeneity analysis the null hypothesis is that the distribution of effect sizes is homogeneous. Within the set of non-pharmaceutical studies, *Q*(df = 49) = 314.930, rejecting the null hypothesis with *p* < .0001. Within the set of pharmaceutical studies, *Q*(df = 46) = 1835.4, rejecting the null hypothesis with *p* < .0001. Due to the rejection of homogeneity for each set of effect sizes, a random-effects model was fitted for each dataset in order to determine the average true effect in the greater population of all possible studies. Results for the various random-effect models are summarized in Table 3. Results for the full study set are available in S1 Results.

**Fig 4:**
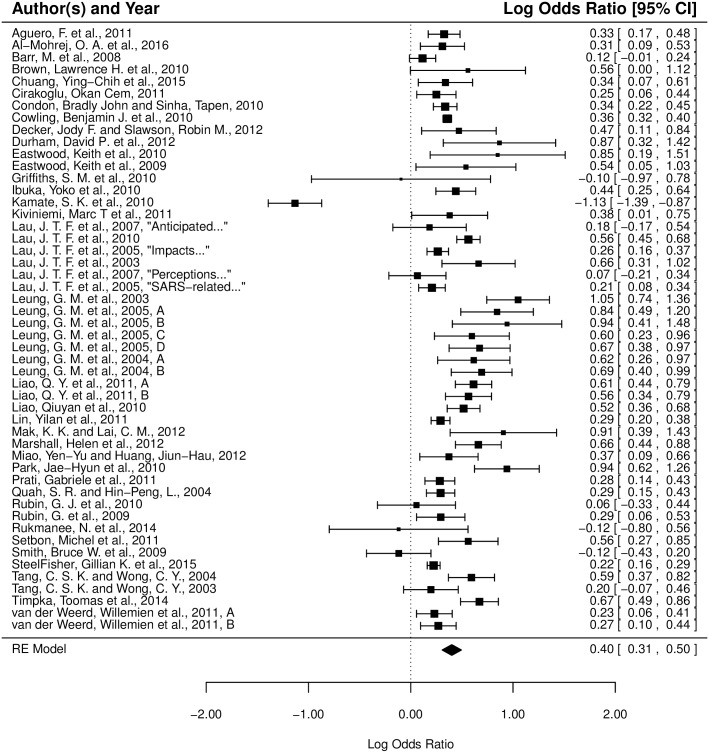
Forest plot of the associations between gender and non-pharmaceutical behaviors. The effect size and confidence interval of each study are indicated by a square and a horizontal line, respectively. The weight of each study in the model is indicated by the size of its square. A log odds ratio of 0, indicated by the dashed reference line, corresponds to no gender difference in behavioral response. Positive log odds ratios correspond to greater behavioral response by females, while negative log odds ratios correspond to greater behavioral response by males. The population mean effect size of the random-effects model incorporating these studies is given by the placement of the diamond, while the horizontal corners of the diamond illustrate the 95% CI of this mean effect size.

The random-effects model for non-pharmaceutical behaviors shows an average true effect of *μ* = 0.402 (95% CI 0.307 to 0.496), implying that women are 49.5% (95% CI 35.9% to 64.2%) more likely than men to adopt/practice non-pharmaceutical behaviors in the general population. The estimated amount of total heterogeneity is *τ*^2^ = 0.093, with *I*^2^ = 92.74% of the total variability in the effect size estimates is due to heterogeneity among the true effects. See Fig 4 for a visualization of the study effect sizes incorporated in the non-pharmaceutical model.

**Fig 5:**
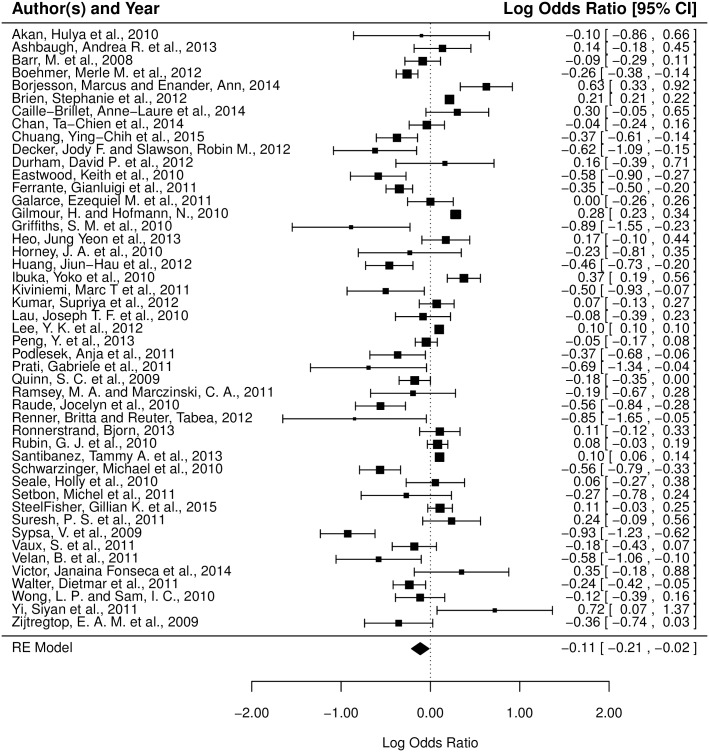
Forest plot of the associations between gender and pharmaceutical behaviors. The effect size and confidence interval of each study are indicated by a square and a horizontal line, respectively. The weight of each study in the model is indicated by the size of its square. A log odds ratio of 0, indicated by the dashed reference line, corresponds to no gender difference in behavioral response. Positive log odds ratios correspond to greater behavioral response by females, while negative log odds ratios correspond to greater behavioral response by males. The population mean effect size of the random-effects model incorporating these studies is given by the placement of the diamond, while the horizontal corners of the diamond illustrate the 95% CI of this mean effect size. Publications with the same author(s) and year of publication are differentiated by the first word of their title. Publications including multiple studies are denoted by labeling the studies A, B, etc.

The random-effects model for pharmaceutical behaviors shows an average true effect of *μ* = −0.114 (95% CI -0.212 to -0.016), implying that men are marginally more likely (specifically, 12.1%, 95% CI 1.6% to 23.6%, more likely) than women to adopt/practice pharmaceutical behaviors in the general population. The estimated amount of total heterogeneity is *τ*^2^ = 0.090, with nearly all of the total variability in the effect size estimates is due to heterogeneity among the true effects (*I*^2^ = 99.78%). See Fig 5 for a visualization of the study effect sizes incorporated in the pharmaceutical model.

In Figs 4 and 5 the distribution of study effect sizes and their corresponding 95% confidence intervals are shown. The dashed reference line, placed at a log odds ratio of 0, corresponds to no gender difference in behavioral response. Studies with squares to the right of the reference line exhibit more female response, while studies with squares to the left of the reference exhibit more male response. The population mean effect size of the random-effects model incorporating these studies is given by the placement of the diamond at the bottom of the figure, while the horizontal corners of the diamond illustrate the 95% CI of this mean effect size.

### Moderator analyses

Mixed-effects models were constructed to test whether any of the heterogeneity among studies exhibited in the random-effects models was due to the influence of moderator variables. For each set of effect sizes publication year, continent, culture (Eastern, Western), country development (developed vs. developing), behavior type (intended, reported, actual), behavior category (preventive, avoidant, management), and overall percentage of respondents adopting/increasing the assessed protective behavior were assessed individually. No significant levels of heterogeneity within the non-pharmaceutical or pharmaceutical study sets were accounted for by any of these moderators. Results for the moderator analysis of the full study set are available in S1 Results.

### Sensitivity analysis

Sensitivity analyses were performed on each set of studies in three ways: by removing effect size outliers, by using the trim-and-fill method, and by including studies whose results weren’t reported on the basis of non-significance. See Table 3 for a summary of sensitivity analysis results. Results for the sensitivity analysis of the full study set are available in S1 Results. For the first sensitivity analysis, only one study [78] from the non-pharmaceutical study set had an effect size greater than 3 standard deviations from the mean and was removed, and none were removed from the pharmaceutical study set. Using the limited non-pharmaceutical dataset, both the fixed- and random-effects models still showed a significant effect size in favor of female behavioral response (${\bar{\theta }}_{w}=0.351$, 95% CI 0.329 to 0.373, and *μ* = 0.423, 95% CI 0.356 to 0.490, respectively).

When the trim-and-fill method was used to augment each set of studies, there were an estimated 9 studies missing from the non-pharmaceutical set and 4 studies missing from the pharmaceutical set. Even though the positive impact of female gender on non-pharmaceutical behavior estimated using the random-effects model is smaller with the missing studies filled in (*μ* = 0.320, 95% CI 0.223 to 0.418), the results still indicate that the effect is statistically significant. When the pharmaceutical set was filled in with the missing studies, the random-effects model showed that the direction of the relationship remained the same but lost significance (*μ* = −0.071, 95% CI -0.175 to 0.034). Funnel plots for each of these study sets are included in S2 Fig.

Four studies [85, 124, 125, 133] failed to report data on the basis of non-significance for all or some behaviors. In the final sensitivity analysis, these unreported results were included as 0s (in other words, neutral effects) in the relevant study set. This method generally leads to more conservative effect size estimates. With the inclusion of the unreported data in this analysis, the direction and significance of the relationship shown by each of the fixed- and random-effects models remained the same for both non-pharmaceutical and pharmaceutical behaviors.

## Discussion

In an effort to elucidate the relationship between gender and health-protective behavior in the general public during respiratory disease epidemics/pandemics, the present meta-analysis was performed to quantitatively combine the results from 85 publications for both non-pharmaceutical and pharmaceutical behaviors.

The result of the random-effects model conclusively shows that women in the general population are 49.5% (95% CI 35.9% to 64.2%) more likely than men to practice and/or increase non-pharmaceutical health-protective behaviors in the context of epidemics/pandemics, with no significant difference found when behavior type or behavior category are included as moderators. This finding is further supported by the results presented in all ten of the studies addressing non-pharmaceutical behaviors for which a direction (but not an effect size) was reported; each showed a positive relationship for the female gender and adoption of the given behaviors. The magnitude of the relationship remained large in each sensitivity analysis.

The random-effects model for pharmaceutical behaviors suggests that men in the general population are slightly (specifically, 12.1%, 95% CI 1.6% to 23.6%) more likely than women to practice and/or increase pharmaceutical health-protective behaviors in the context of epidemics/pandemics. However, nearly all of the variability in the effect size estimates was due to heterogeneity among the true effects, none of which could be accounted for by including moderators. Furthermore, the addition of studies based on the trim-and-fill method rendered the observed relationship non-significant. Although the study design of this meta-analysis limits the danger of publication bias influencing the results, the random-effects model using the filled-in study set implies that if publication bias is indeed present, then it has falsely skewed the results in favor of significance. In spite of this finding, the results of the other sensitivity analyses still favor a mildly positive relationship between male gender and uptake of pharmaceutical behaviors. Of the studies addressing pharmaceutical behaviors for which a direction (but not an effect size) was reported males tended to exhibit more behavioral response than females, but this observed relationship has little value given the small sample size (5 studies).

While no significant moderators were found for the non-pharmaceutical or pharmaceutical study sets, the set of moderators tested in this study was not comprehensive. There are a variety of study-level differences that were not tested as moderators in this analysis, including perceived severity of the disease, demographic characteristics of the study sample other than gender (including mean age, income, education level, minority status, and risk status), and whether the response addressed absolute uptake or increase in uptake of behavior. While the perception of the severity of a disease likely impacts health-protective behavior and may act as a moderator of the relationship we address, we do not have adequate data to create a metric for perceived severity for the publications that did not explicitly report it. Data on the severity of a given epidemic/pandemic respiratory disease outbreak are available in terms of case counts and mortality rates, but data on perceived severity are not so easily obtained. Perceived severity may depend on the proximity of the study population to high-risk areas, news media focus and tone, phase of epidemic/pandemic in which surveys/questionnaires were administered, and a host of other intangibles that extend beyond the scope of this analysis. Similarly, while study-level demographic differences (i.e., one study administering questionnaires to mostly young people, another to mostly old people) could have an effect, there are not enough studies coming from heavily age-skewed demographic groups to make claims about the impact of age (or other demographic differences in study populations) on the relationship between gender and health-protective behavior.

Further research could possibly elucidate which, if any, of these unexplored study-level differences moderate the relationship between gender and health-protective behavior. It also is possible that including a greater number of studies across a wider range of countries could elucidate a moderator effect that was simply too weak to be found in the present meta-analysis. However, a concern with assessing too wide a range of potential moderators is the possibility of falsely significant results appearing simply due to over-testing. The moderators addressed in this study balanced exploration and plausibility, while taking feasibility of moderator calculation into consideration.

This study focused on the influence of gender on health-protective behavior. Many other geographic, demographic, and psychological factors have been shown to influence the uptake of health-protective behavior during respiratory epidemics/pandemics. Further study could focus on meta-analytically analyzing these other possible behavioral determinants (e.g., age, income, phase of epidemic/pandemic, country development) to develop a fuller understanding of the health-protective behavior of the general public during epidemics/pandemics.

A wide array of health-protective behaviors were considered in this analysis. It may be argued that this leads to the problem of comparing apples and oranges, but the separation of the study sets into pharmaceutical and non-pharmaceutical groups mitigates this issue. In the case of non-pharmaceutical behaviors, a particular action is not as important to policy makers as a general behavioral trend, upon which health campaigns can base targeting and advertising. Similarly, mathematical disease models including behavior can use these results to parameterize demographic-based model values. In summary, the present study quantitatively suggests that gender influences health-protective behavioral response in the general public, with females being more likely to adopt/increase non-pharmaceutical behaviors and males being more likely to adopt/increase pharmaceutical behaviors. Additional research into moderators of this relationship might help to understand the contexts in which it is attenuated or strengthened. Additionally, a quantitative analysis of other determinants of health-protective behavior could further assist policy makers and model builders.

## Supporting Information

S1 Code R code. This .R file contains the script used to run the meta analysis in R.
(R) Click here for additional data file.

S2 Code Python code. This .py file contains the script used to calculate weighted averages of multiple effect sizes.
(PY) Click here for additional data file.

S1 Data Final model datasets. This .xlsx file contains a sheet for each of the following: the set of data for included studies addressing pharmaceutical behaviors, the set of data for included studies addressing non-pharmaceutical behaviors, the set of data for included studies addressing all behaviors, and analogous datasets including non-significant unreported data as 0.
(XLSX)

S1 Table Web of Science and PubMed queries. This PDF file shows a table of the explicit search terms entered when querying the Web of Science and PubMed database. The timespan specified for all searches was 2002 to present.
(PDF)

S2 Table Qualitative and quantitative study data. This .xlsx file contains sheets for the following: the qualitative data on all included studies, the data extracted from each study and used in model construction, and the direction of the relationship for each study for which an explicit effect size could not be calculated.
(XLSX)

S1 Fig PRISMA checklist. Page numbers of all PRISMA-required information are provided.
(PDF)

S2 Fig Forest plots showing imputed study values for pharmaceutical and non-pharmaceutical study sets. Black circles correspond to actual studies, white circles correspond to imputed study values. The vertical reference line indicates the mean true effect of the random-effects model including both actual and imputed study values.
(PDF)

S1 Results Results for full study set. A PDF file giving fixed-, random-, and mixed-effects model results for the set of all 88 included study populations. Sensitivity analysis results are also shown.
(PDF)
