# Supplementary material for: A Meta-Analysis of the Association between Gender and Protective Behaviors in Response to Respiratory Epidemics and Pandemics
Source: PLoS One. 2016 Oct 21;11(10):e0164541. doi: 10.1371/journal.pone.0164541 (PMC5074573; doi:10.1371/journal.pone.0164541)
Supplement: S1 Table — This PDF file shows a table of the explicit search terms entered when querying the Web of Science and PubMed database. The timespan specified for all searches was 2002 to present. (PDF) [file pone.0164541.s004.pdf]

Table S1: **Web of Science and PubMed queries.** The explicit search terms entered when querying the Web of Science and PubMed database. The timespan specified for all searches was 2002 to present (with present being the date of the query). Note that later queries were performed in order to include MERS in the analysis.

**Search terms**	**Date**	**Results**
**Web of Science searches**		
TOPIC: (pandemic OR epidemic) AND TOPIC: (sex OR gender OR demographic$) AND TOPIC: (behavio* OR response OR pharmaceutical OR non-pharmaceutical OR intervention$ OR mitigation$) AND TOPIC: (h1n1 OR h5n1 OR sars OR "avian influenza" OR "avian flu" OR "bird flu" OR "swine flu")	8/25/2015	242
TOPIC: (sars OR "severe acute respiratory syndrome") AND TOPIC: (behavio* OR "face mask" OR "hand washing" OR quarantine OR response OR pharmaceutical OR non-pharmaceutical OR intervention$ OR mitigation$) AND TOPIC: (sex OR gender OR demographic$ OR determinant$)	8/27/2015	153
TOPIC: (pandemic$ OR epidemic$) AND TOPIC: (age) AND TOPIC: (behavio* OR "face mask" OR "hand washing" OR quarantine OR intervention) AND TOPIC: (h1n1 OR h5n1 OR sars OR "avian influenza" OR "avian flu" OR "bird flu" OR "swine flu")	8/27/2015	438
TOPIC: (pandemic OR epidemic) AND TOPIC: (sex OR gender OR demographic$) AND TOPIC: (behavio* OR response OR pharmaceutical OR non-pharmaceutical OR intervention$ OR mitigation$) AND TOPIC: (mers OR "middle east respiratory syndrome")	6/14/2016	1
TOPIC: (mers OR "middle east respiratory syndrome") AND TOPIC: (behavio* OR "face mask" OR "hand washing" OR quarantine OR response OR pharmaceutical OR non-pharmaceutical OR intervention$ OR mitigation$) AND TOPIC: (sex OR gender OR demographic$ OR determinant$)	6/14/2016	116
TOPIC: (pandemic$ OR epidemic$) AND TOPIC: (age) AND TOPIC: (behavio* OR "face mask" OR "hand washing" OR quarantine OR intervention) AND TOPIC: (mers OR "middle east respiratory syndrome")	6/14/2016	3

**Search terms**	**Date**	**Results**
**PubMed searches**		
((((pandemic[Title/Abstract] OR epidemic[Title/Abstract])) AND (sex[Title/Abstract] OR gender[Title/Abstract] OR demographic*[Title/Abstract])) AND (behavio*[Title/Abstract] OR response[Title/Abstract] OR pharmaceutical[Title/Abstract] OR non-pharmaceutical[Title/Abstract] OR intervention*[Title/Abstract] OR mitigation*[Title/Abstract])) AND (h1n1[Title/Abstract] OR h5n1[Title/Abstract] OR sars[Title/Abstract] OR “avian influenza”[Title/Abstract] OR “avian flu”[Title/Abstract] OR “bird flu”[Title/Abstract] OR “swine flu”[Title/Abstract])	8/31/15	165
(((sars[Title/Abstract] OR “severe acute respiratory syndrome”[Title/Abstract])) AND (behavio*[Title/Abstract] OR “face mask”[Title/Abstract] OR “hand washing”[Title/Abstract] OR quarantine[Title/Abstract] OR response[Title/Abstract] OR pharmaceutical[Title/Abstract] OR non-pharmaceutical[Title/Abstract] OR intervention*[Title/Abstract] OR mitigation*[Title/Abstract])) AND (sex[Title/Abstract] OR gender[Title/Abstract] OR demographic*[Title/Abstract] OR determinant*[Title/Abstract])	8/31/15	63
((((pandemic[Title/Abstract] OR epidemic[Title/Abstract])) AND age[Title/Abstract]) AND (behavio*[Title/Abstract] OR “face mask”[Title/Abstract] OR “hand washing”[Title/Abstract] OR quarantine[Title/Abstract] OR intervention[Title/Abstract])) AND (h1n1[Title/Abstract] OR h5n1[Title/Abstract] OR sars[Title/Abstract] OR “avian influenza”[Title/Abstract] OR “avian flu”[Title/Abstract] OR “bird flu”[Title/Abstract] OR “swine flu”[Title/Abstract])	8/31/15	142
((((pandemic[Title/Abstract] OR epidemic[Title/Abstract])) AND (sex[Title/Abstract] OR gender[Title/Abstract] OR demographic*[Title/Abstract])) AND (behavio*[Title/Abstract] OR response[Title/Abstract] OR pharmaceutical[Title/Abstract] OR non-pharmaceutical[Title/Abstract] OR intervention*[Title/Abstract] OR mitigation*[Title/Abstract])) AND (mers[Title/Abstract] OR “middle east respiratory syndrome”[Title/Abstract])	6/14/2016	0
(((mers[Title/Abstract] OR “middle east respiratory syndrome”[Title/Abstract])) AND (behavio*[Title/Abstract] OR “face mask”[Title/Abstract] OR “hand washing”[Title/Abstract] OR quarantine[Title/Abstract] OR response[Title/Abstract] OR pharmaceutical[Title/Abstract] OR non-pharmaceutical[Title/Abstract] OR intervention*[Title/Abstract] OR mitigation*[Title/Abstract])) AND (sex[Title/Abstract] OR gender[Title/Abstract] OR demographic*[Title/Abstract] OR determinant*[Title/Abstract])	6/14/2016	23
((((pandemic[Title/Abstract] OR epidemic[Title/Abstract])) AND age[Title/Abstract]) AND (behavio*[Title/Abstract] OR “face mask”[Title/Abstract] OR “hand washing”[Title/Abstract] OR quarantine[Title/Abstract] OR intervention[Title/Abstract])) AND (mers[Title/Abstract] OR “middle east respiratory syndrome”[Title/Abstract])	6/14/2016	0
