# Supplementary material for: A Meta-Analysis of the Association between Gender and Protective Behaviors in Response to Respiratory Epidemics and Pandemics
Source: PLoS One. 2016 Oct 21;11(10):e0164541. doi: 10.1371/journal.pone.0164541 (PMC5074573; doi:10.1371/journal.pone.0164541)
Supplement: S2 Fig — Black circles correspond to actual studies, white circles correspond to imputed study values. The vertical reference line indicates the mean true effect of the random-effects model including both actual and imputed study values. (PDF) [file pone.0164541.s007.pdf]

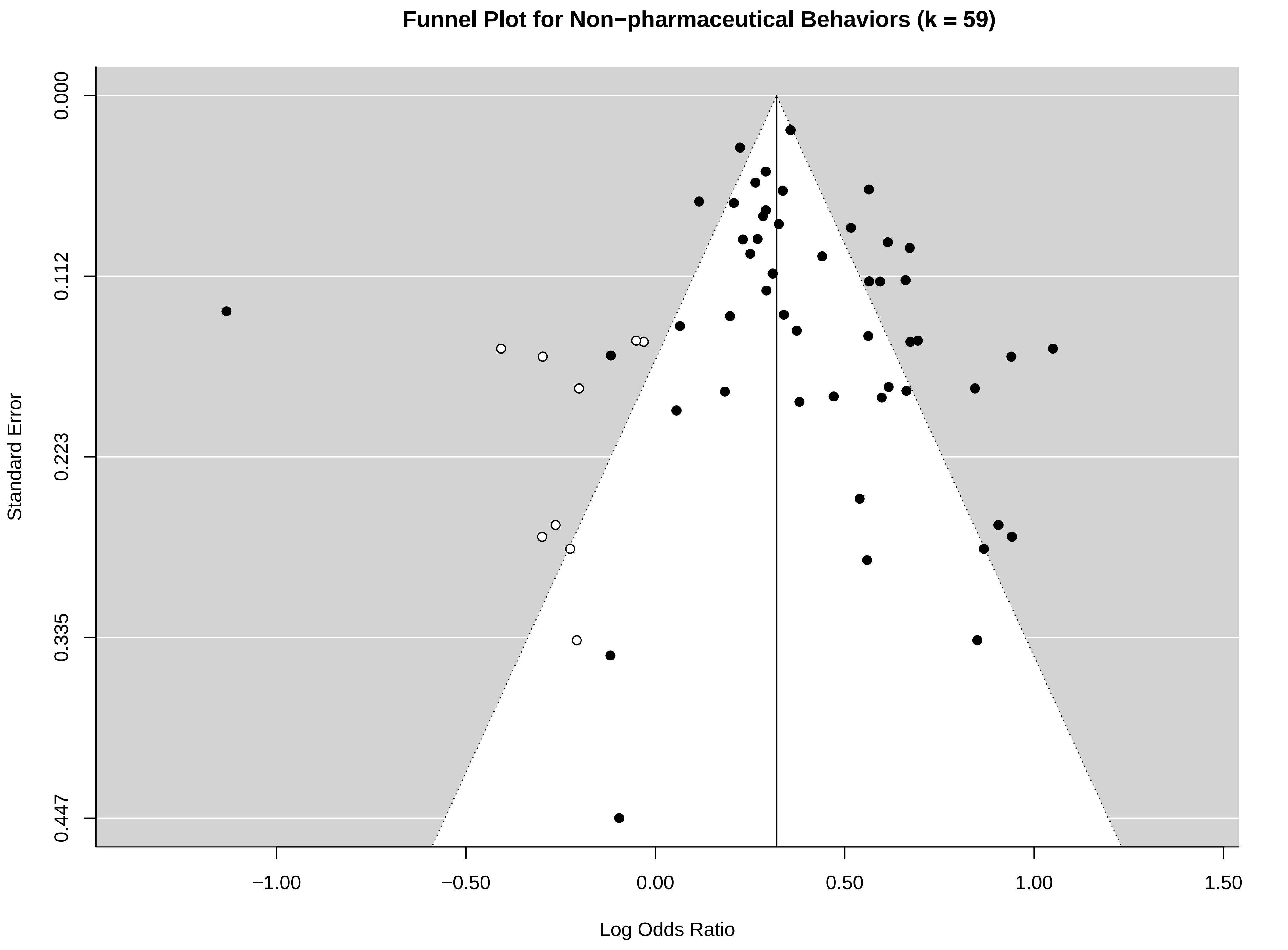
Funnel Plot for Non−pharmaceutical Behaviors (k = 59)
Standard Error
0.000
0.112
0.223
0.335
0.447
−1.00
−0.50
0.00
0.50
1.00
1.50
Log Odds Ratio

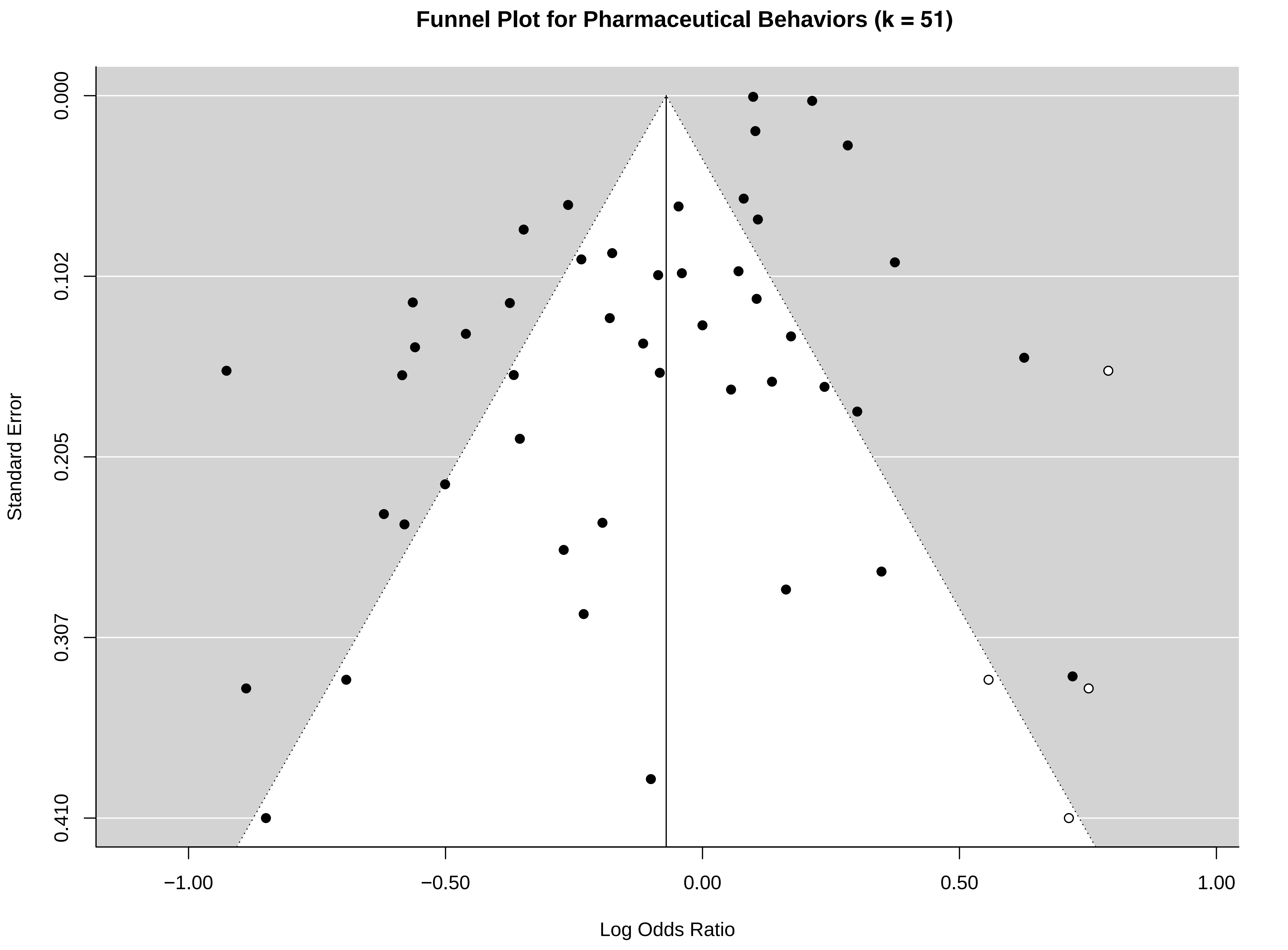
Funnel Plot for Pharmaceutical Behaviors (k = 51)
Standard Error
0.000
0.102
0.205
0.307
0.410
Log Odds Ratio
−1.00
−0.50
0.00
0.50
1.00

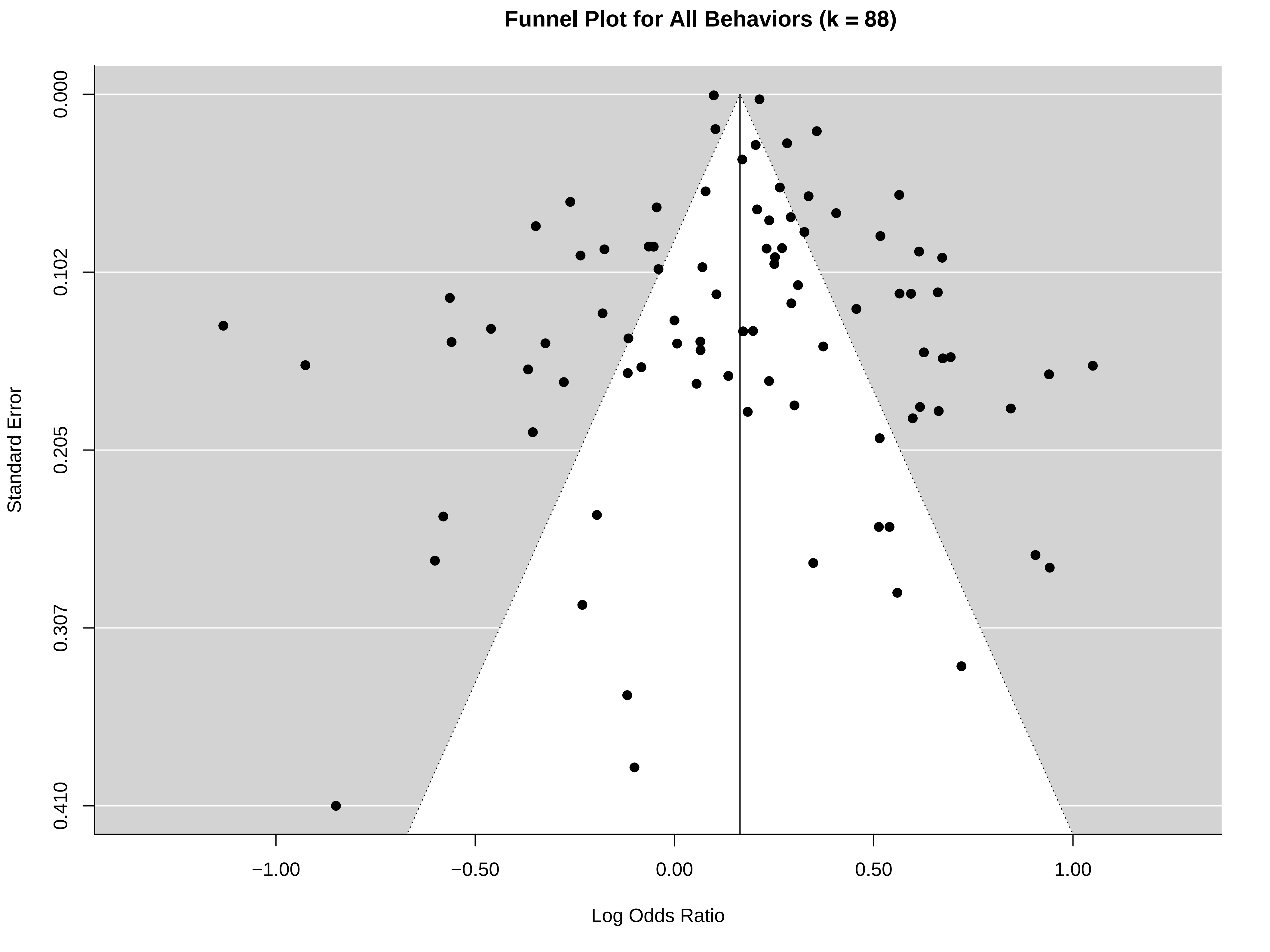

Funnel Plot for All Behaviors (k = 88)
Standard Error
0.000
0.102
0.205
0.307
0.410
−1.00
−0.50
0.00
0.50
1.00
Log Odds Ratio
