# Supplementary material for: A Meta-Analysis of the Association between Gender and Protective Behaviors in Response to Respiratory Epidemics and Pandemics
Source: PLoS One. 2016 Oct 21;11(10):e0164541. doi: 10.1371/journal.pone.0164541 (PMC5074573; doi:10.1371/journal.pone.0164541)
Supplement: S1 Results — A PDF file giving fixed-, random-, and mixed-effects model results for the set of all 88 included study populations. Sensitivity analysis results are also shown. (PDF) [file pone.0164541.s008.pdf]

Full study set results

The fixed-effects model for the full study set indicated the overall effect size when considering all 88 studies was $\bar{\theta}_w = 0.105$ (95% CI 0.103 to 0.106). For homogeneity analysis the null hypothesis is that the distribution of effect sizes is homogeneous. Across all studies, $Q(\text{df} = 87) = 2498.873$, rejecting the null hypothesis with $p < .0001$. Due to the rejection of homogeneity, a random-effects model was fitted in order to determine the average true effect in the greater population of all possible studies. The random-effects model for all behaviors shows an average true effect of $\mu = 0.164$ (95% CI 0.079 to 0.250), implying that women are overall slightly (about 1.2 times) more likely than men to adopt/practice any health-protective behaviors. The estimated amount of total heterogeneity when the random-effects model includes all behaviors is $\tau^2 = 0.145$. Nearly all of the total variability is due to heterogeneity ($I^2 = 99.76\%$).

Mixed-effects models were constructed to test whether any of the heterogeneity among studies exhibited in the random-effects models was due to the influence of moderator variables. Pharmaceutical/non-pharmaceutical status, publication year, continent, culture (Eastern, Western), country development (developed vs. developing), behavior type (intended, reported, actual), behavior category (preventive, avoidant, management), and overall percentage of respondents adopting/increasing the assessed protective behavior were assessed individually.

Upon testing each moderator within the full study set, pharmaceutical/non-pharmaceutical status among all behaviors accounted for a significant amount of heterogeneity. The estimated amount of residual heterogeneity τ^2 is equal to 0.094 (95% CI 0.069 to 0.150), suggesting that approximately $(0.145 - 0.094)/0.145 = 35\%$ of the total amount of heterogeneity can be accounted for by including pharmaceutical/non-pharmaceutical status in the model for all behaviors. The test for residual heterogeneity is significant ($Q_{res}(\text{df} = 85) = 2099.8$, $p < .0001$), implying that there may still be other moderators not considered in the model that influence gender differences in behavioral response. The results indicate that $\beta_0 = 0.417$ (95% CI 0.308 to 0.526) is the estimated average log odds ratio for studies addressing only non-pharmaceutical behaviors, while $\beta_1 = -0.502$ (95% CI -0.661 to -0.343) and $\beta_2 = -0.318$ (95% CI -0.510 to -0.126) estimate how much smaller the average log odds ratios are when studies address only pharmaceutical behaviors and both types of behavior, respectively.

When considered on its own, culture accounted for $(0.145 - 0.134)/0.145 = 8\%$ of the total amount of heterogeneity of the full study set. Eastern cultures had log odds ratio of $\beta_0 = 0.293$ (95% CI 0.167 to 0.419), with $\beta_1 = -0.225$ (95% CI -0.392 to -0.058) estimating how much smaller the average log odds ratio is when only studies from Western countries are considered. This finding suggests that overall Eastern countries tend to have a higher relative rate of female to male behavioral response levels. While the direction of this relationship is the same for the non-pharmaceutical

and pharmaceutical datasets, it lacks significance in these reduced study sets. Furthermore, culture does not remain significant when included in a regression model for the full study set including both pharmaceutical/non-pharmaceutical status and culture.

Sensitivity analyses were performed in three ways: by removing effect size outliers, by using the trim-and-fill method, and by including studies whose results weren't reported on the basis of non-significance. For the first sensitivity analysis, no studies had an effect size greater than 3 standard deviations from the mean and thus none were removed. When the trim-and-fill method, no additional study values were necessary in the full behavioral study set. Four studies failed to report data on the basis of non-significance for all or some behaviors. In the final sensitivity analysis, these unreported results were included as 0s (in other words, neutral effects) in the relevant study set. This method generally leads to more conservative effect size estimates. With the inclusion of the unreported data in this analysis, the direction and significance of the relationship shown by the fixed- and random-effects models remained the same.

Table S1: **Fixed- and random-effects model results.** Includes the full study set and the three corresponding sensitivity analysis sets.

	**Full study set**			
	Original (k = 88)	Outlier removal (k = 88)	Trim-and-fill (k = 88)	With unreported (k = 90)
**Fixed-effects model**				
$\bar{\theta}_w$ *(95% CI)*	0.105 (0.103 to 0.106)	**	*	0.105 (0.103 to 0.106)
Q *(p-val)*	2498.873 (<0.0001)	**	*	2472.861 (<0.0001)
**Random-effects model**				
μ *(95% CI)*	0.164 (0.079 to 0.250)	**	**	0.151 (0.070 to 0.234)
τ^2 *(95% CI)*	0.145 (0.109 to 0.222)	**	**	0.136 (0.102 to 0.209)
I^2 *(95% CI)*	99.76% (99.68 to 99.84%)	**	**	99.74% (99.65 to 99.83%)

$\bar{\theta}_w$: Average true effect of the set of studies included in the analysis.
Q-statistic: Measure of heterogeneity. Calculated as the weighted sum of squared differences between individual study effects and the pooled effect across studies, with the weights being those used in the pooling method chi-square statistic with k-1 degrees of freedom μ: Average true population effect size.
τ^2: Total amount of heterogeneity among the true effects.
I^2: Percent of the total variability in effect size estimates due to heterogeneity among the true effects.
* Trim-and fill analysis results for fixed-effects model not relevant due to non-homogeneous effect size distribution.
** Results not shown because study set unchanged from original.
